# Fine-Tuning an ISFET-Based Dual pH–Total Alkalinity Sensor for Operation in Seawater Using an Adjustable, Suspended Anode

**DOI:** 10.1021/acssensors.5c02765

**Published:** 2025-09-28

**Authors:** Ellen M. Briggs, Todd R. Martz

**Affiliations:** Scripps Institution of Oceanography, 8784 University of California San Diego, La Jolla, California 92037, United States

**Keywords:** seawater, pH, total alkalinity, in situ sensor, ISFET

## Abstract

In this study, we present a re-envisioned design of an ion-sensitive field-effect transistor (ISFET)-based solid-state sensor for measuring pH and total alkalinity of seawater with electrolytically generated titrant. The original design requires either custom nanofabrication of ISFET wafers or back-end processing of fully fabricated ISFETs. Instead, we assembled all “off-the-shelf” parts to demonstrate the same measurement principle but with the titrant-generating electrode suspended orthogonal to the pH-sensing region of the ISFET rather than being physically deposited on the face of the chip. This offers many benefits including (1) avoiding disruption the pH functionality of the ISFET; (2) enabling the assessment of anode–gate distance sensitivity from a single ISFET; and (3) exploration of different electrode geometries and composition without complex ISFET fabrication. In this study, we analyzed the sensitivity of the A_T_ measurement to the spacing of the titrant-generating electrode (anode) to the pH-sensing region of the ISFET (gate) as well as sensitivity to the electric current applied to the anode at three seawater A_T_ compositions. The greatest sensor resolution in seawater was achieved when operating at a low anode current (5 μA) and greater anode–gate distance (∼150 to 200 μm) for the suspended anode configuration. The suspended anode configuration also showed an improvement in equivalence point determination with sharper inflections relative to the on-chip anode configuration. By averaging (*N* = 5), a sensor error of 2.85 μmol kg^–1^ was achieved over 131 measurements.

## Graphical abstract

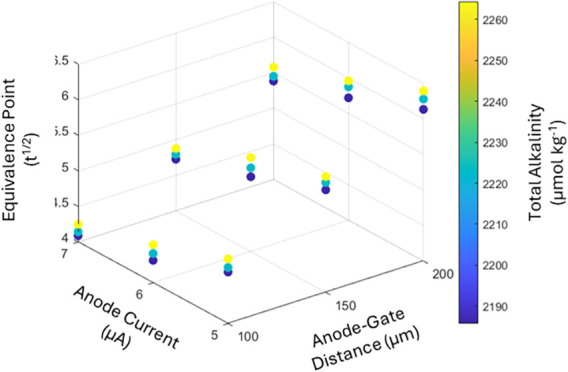

The ocean plays an integral role in the global carbon cycle. As more anthropogenic CO_2_ is released to the atmosphere, it is essential to understand and quantify the impact on the ocean’s role in the uptake, transfer, and transformation of carbon as well as cascading effects on biogeochemical (BGC) processes.^1,2^ Discrete sampling is often insufficient to resolve the many important features of marine BGC cycles and subsequent feedbacks that occur at much shorter times and larger spatial scales. One particular challenge has been the ability to collect measurements of relevant ocean carbon system parameters in remote, less accessible locations or from autonomous platforms.^3–7^

Direct, in situ measurements of ocean inorganic carbon chemistry including pH, total dissolved inorganic carbon (C_T_), partial pressure of CO_2_ (pCO_2_), and total alkalinity (A_T_) are desirable. Two of the four parameters (pH, C_T_, pCO_2_, and A_T_) are required to fully constrain the aqueous carbon dioxide system and combinations of either pH or pCO_2_ with A_T_ or C_T_ are preferred because they are more distinctively altered by primary production, gas exchange, and calcification.^8,9^ However, A_T_ and C_T_ are the most difficult parameters to measure in situ, and current chemical sensor technology does not fulfill the low power, compact design, rapid sampling, and stability requirements for use on autonomous platforms. While it is possible to colocate separate autonomous sensors for pH and pCO_2_ to obtain near-simultaneous measurements, this combination of variables is undesirable due to nature of error propagations through the CO_2_ system calculations.^10,11^

Seawater total alkalinity (the excess of proton acceptors over proton donors)^12^ traditionally involves determination by acid–base titration which is difficult to automate. Globally, seawater A_T_ spans a relatively narrow range ∼2100 to 2500 μmol kg^–1^. In the open surface ocean, A_T_ is dominantly controlled by simple physical processes (e.g., precipitation, evaporation) and can often be estimated rather accurately from measurements of temperature and salinity.^13,14^ Empirical relationships are also used to estimate A_T_ in the interior ocean.^15,16^ However, empirical estimates fail under various conditions, including dynamic environments both at depth and at the ocean surface where processes such as freshwater input and calcium carbonate formation and dissolution lead to nonconservative behavior in A_T_. Coccolithophore blooms, sediment porewaters, coral reefs, river mouths, mangroves, and coastal shelves are general examples where nonconservative behavior is known to occur.^17–19^

An emerging field of research focusing on marine-based carbon dioxide removal (mCDR) strategies, such as alkalinity enhancement, nutrient fertilization, and various electrochemical approaches, would benefit from simplified analysis of seawater alkalinity.^20^ If it is implemented, mCDR will require monitoring and verification strategies to quantify the efficacy and durability of carbon storage as well as to identify environmental and societal impacts. This requires a robust, autonomous, and full-carbon sensor compatible with platforms capable of monitoring at various spatiotemporal scales.

Briggs et al.^21,22^ demonstrated the proof of concept and preliminary field deployment of a novel, solid-state, reagentless sensor capable of rapid and near-simultaneous measurement (<60 s) of pH and A_T_. This prototype pH–A_T_ sensor utilizes ion-sensitive field-effect transistor (ISFET) pH-sensing technology coupled with a coulometric diffusion titration (CDT) technique to additionally measure A_T_. The ability to measure two carbon system parameters on similar time scales on one solid-state chip sets this sensor apart from currently available observing technologies. With a small footprint, no external reagents, and low power consumption, this sensor meets many of the demands required for autonomous platforms.

ISFET pH sensor technology is now mature.^23^ For instance, Honeywell has commercialized the DuraFET sensor, which is a pH-sensing ISFET-based combination electrode currently used in industrial as well as oceanographic applications.^24–28^ As solid-state pH sensors, ISFETs, offer advantages relative to glass electrodes including reduced drift as well as the ruggedness required for in situ, autonomous use. ISFET pH sensor technology has also been adapted for higher-pressure applications, enabling integration onto profiling floats (Deep Sea DuraFET, DSD) for autonomous monitoring of seawater pH in the upper 2000 m.^25^

By integrating a coulometric actuator device with an ISFET, a microscale acid–base titration can be simultaneously executed and measured on a single ISFET chip to measure A_T_ (or total acidity),^29–34^ thus converting the pH sensor into a dual pH and A_T_ sensor. The original intended application of the ISFET-based titrator was for the food and beverage industry,^34^ and the concept was later developed and demonstrated in seawater.^21,22^ Titrant, H^+^, is generated through the electrolysis of water by applying an anodic current pulse to an actuator electrode, with respect to a counter electrode in an analyte solution.

$$a\mathrm{node}:2H_{2}O\to 4H^{+}+4e^{-}+O_{2}$$

$$c\mathrm{athode}:2H_{2}O+2e^{-}\to 2{\mathrm{OH}}^{-}+H_{2}$$

With the CDT method, analyte concentration is expressed as a function of the square root of time according to the Sand equation

$$t_{sand}={\left[\frac{C_{B,bulk}F\sqrt{\pi D_{B}}}{2j_{c}}\right]}^{2}$$

where *C*_B_ is the analyte concentration (i.e., A_T_), *F* is the Faraday constant, *D*_B_ is the diffusion coefficient of the analyte, and *j*_c_ is the current density applied to the actuator electrode. The pH is rapidly measured (Figure 1) while electrolytically generated protons neutralize the analyte. In seawater, the square root of the time required to reach the second inflection (Figure 1, blue line) is linearly proportional to A_T_.^22^

**Figure 1:**
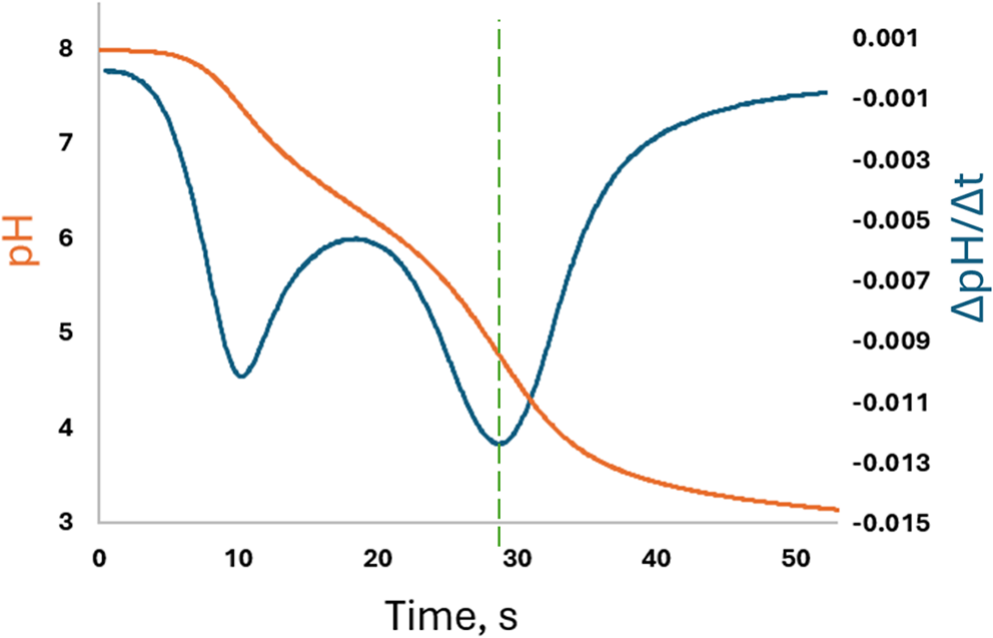
Example titration curve (pH, orange) and corresponding first derivative (blue) in seawater for the re-envisioned pH–A_T_ sensor with suspended anode. The 2nd inflection corresponds to the ep, as indicated by the green dashed line.

In the original design, the actuator electrode is deposited directly on the surface of the ISFET near the gate (pH-sensitive region of the chip) in order to achieve the necessary proximity of the pH sensor and the electrolytically generated H^+^.^33^ While this approach has the benefit of a compact design, there are a few drawbacks. First, Honeywell was uninterested in performing small batch custom ISFET fabrication so individual fully fabricated ISFET chips were subjected to “back-end processing techniques” (i.e., manual post-processing finishing steps outside of standard CMOS or complementary metal-oxide semiconductor fabrication) in order to add the actuator electrode (anode) which is labor-intensive and challenging to scale from a manufacturing standpoint. There are also limitations to what fabrication protocols can be utilized on a fully processed ISFET such as chemicals that may corrode the gate oxide or elevated temperatures (>350 °C) that will melt metal contact pads. If any trace material is unintentionally deposited on the gate of the ISFET, the Nernstian response or pH sensitivity would be altered. Finally, although never tested until this work, an ISFET-facing suspended anode, as opposed to a die-mounted adjacent anode, may provide geometrical advantages including a more uniform proton diffusion front that more closely obeys the ideal diffusion conditions represented by the Sand equation.

It is feasible to use a different ISFET fabrication facility capable of including electrode deposition steps during wafer fabrication. This is currently being pursued but is outside the scope of the work presented here. To date, this approach has proven to be expensive in terms of both cost and time. The novel approach pursued in this work is the integration of the titrant-generating electrode into the mechanical housing that seals the chip, which can be adapted to various ISFET sources.

In this study, we report the results of using “off-the-shelf” parts assembled to measure both pH and A_T_ utilizing ISFET technology and the CDT A_T_ approach. We used a commercially available “stock” DuraFET coupled with a platinum disc electrode and a micrometer-driven translational stage to suspend the titrant-generating electrode vertically above the DuraFET pH-sensing gate to provide A_T_ determination in addition to pH measurement. This assembly enabled the determination of the A_T_ equivalence point (ep) sensitivity to the anode current and anode–gate distance, which can then be used in future device design to optimize the range and resolution of the sensor. The optimal “sweet spot” had not yet been determined for typical seawater range or the more extreme values observed in, e.g., mangroves, porewater, and river mouths.

## Methods

A stock DuraFET pH probe (Honeywell) was installed in a custom 3D printed cell with the face of the ISFET adjusted to be orthogonal to the z-plane with a leveling device (Figure 2). A 1 mm platinum disc electrode serving as the titrant-generating anode (ET075-1, eDAQ) was attached to a translational stage coupled to a micrometer (MS1S/M, ThorLabs). The micrometer offers 10 μm of translation per gradient and was manually adjusted during the experiments. The platinum electrode was secured with a 3D printed sleeve that was mounted onto the translational stage, which was then mounted vertically above (*z*-plane) the DuraFET in the custom cell. A 316 stainless steel bolt was used as the cathode.

**Figure 2:**
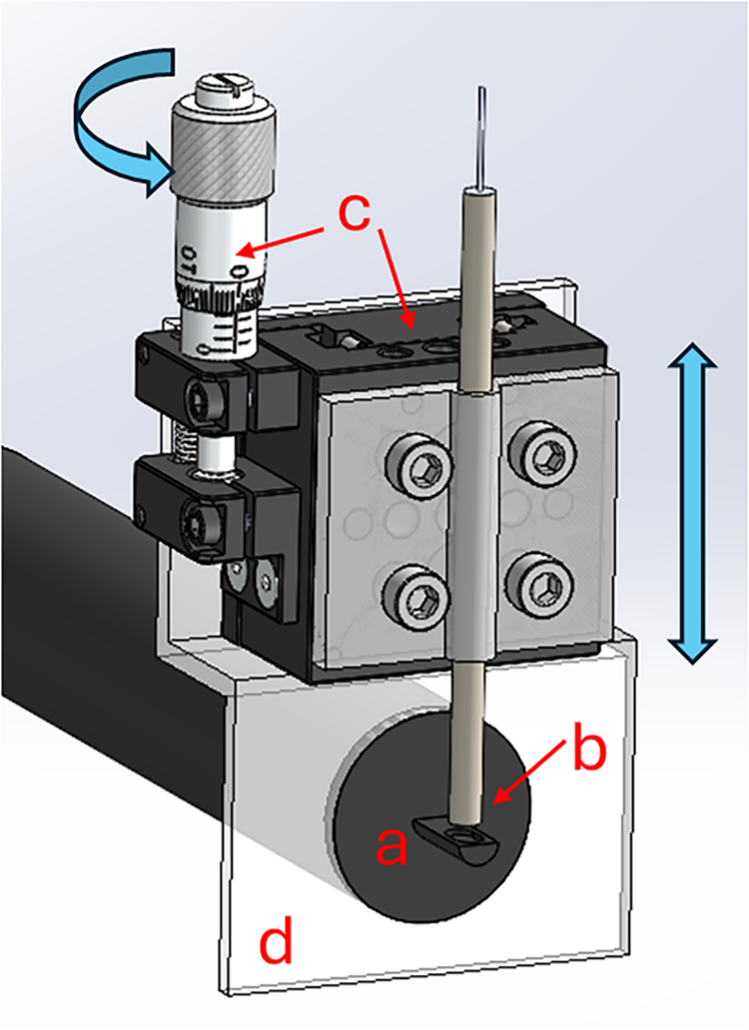
Assembly showing off-the-shelf parts for measuring pH and A_T_ including a DuraFET pH sensor (a), a platinum disc electrode in a PEEK sleeve (b), and a micrometer adjustable translational stage (c), all of which were integrated into a custom 3D printed cell (cutaway shown by (d)) that was filled with seawater. By turning the micrometer, the translation stage moves the secured platinum electrode orthogonally with respect to the ISFET gate with 10 μm graduations.

To open up the flushable area between the disc electrode and DuraFET, the PEEK sleeve of the disc electrode was sanded by hand to a conical shape. The platinum electrode was additionally polished prior to assembly with 3 polishing solutions with diamond particle sizes of 15, 3, and 1 μm on a nylon polishing disc (MF-2060, BASi). After each polishing step, the platinum electrode was rinsed thoroughly with acetone and gently wiped dry with an acetone-soaked Kimwipe.

The optimal height of the anode was determined by running titrations in CO_2_-certified reference materials (Scripps Institution of Oceanography) while tuning the anode position to achieve approximately 30 s to reach the carbonate ep. This time–height selection was based on the previous work with the anode deposited directly on the surface of the ISFET.^22^ The absolute height of the anode was then determined by lowering the anode until it made contact with the ISFET at the completion of the experiment to avoid any damage to the DuraFET.

To determine the sensitivity of the ISFET-based CDT approach to anode current and anode–gate distance, a series of titrations were performed at 5, 6, and 7 μA (anode current) and 100, 150, and 200 μm (anode–gate distance) in seawater with 3 known A_T_ values (2264, 2223, and 2186 μmol kg^–1^) adjusted by dilution with weighed DI water additions. This generated 27 permutations, with 5 titrations collected at each step for a total of 135 measurements. Linear regressions were generated to assess the sensitivity of the ep time to the anode current and the anode–gate distance. Additionally, linear regressions of the standard dilutions for each of the configurations were used as calibration curves to translate ep to A_T_ units. The order in which the configurations were tested was randomized (with the exception of known A_T_ values, which were only decreasing by dilution) so that any possible drift would change the linearity of the slope instead of showing up as just a change in the slope. From previous experiments performing standard dilutions, we have not observed a salinity-dependent effect on A_T_ ep determination over the relatively narrow range of open ocean salinity, and thus, we did not try to control salinity to a static value during this set of experiments.

A submersible pump (SBE 5 M Mini Submersible Pump, SeaBird) with a 25 mL s^–1^ flow rate was used to flush the anode–gate region between measurements. The pump ran for 3 min at the start of a new configuration and 2 min between each replicate. Pump ON-time was made sufficiently long to ensure the small anode–gate gap was fully flushed and to dislodge any bubbles that may form during the electrolysis of water which disrupt the CDT method. The vertical gap between the suspended anode and the ISFET occupied a volume of 0.08–0.2 μL corresponding to an anode–gate distance of 100–200 μm, so very little volume was required to flush and introduce new samples. However, the pump was only pointed at the gap and much of the flow went around the gap rather than through it requiring longer flushing times. From past experiences, whenever the sensor was idle where the anode current was set to zero for longer durations (i.e., >5 min), the first titration is left out of calculations because there is a “burn-in” time where the first measurement is often an outlier. To remain as consistent as possible across the data set, the pump OFF-time was minimized between configurations. All of the experiments were run at ambient temperature in a climate-controlled laboratory.

## Results

Out of the 135 measurements, 4 were flagged (corresponding to the configuration: 7 μA, 100 μm, 2223 μmol kg^–1^) as possible disruption due to bubble formation identified as a sizable reduction in ep time relative to the first measurement. When processing all of the data without these 4 data points, the linear regressions substantially improved, providing greater confidence in flagging these data points and leaving them out of the sensitivity analyses reported here. It is likely that some of the other outliers in the remaining 131 measurements could be flagged as possible bubble disruption; however, there was not as conclusive evidence to provide justification in leaving them out of this analysis. The full table of configurations, ep(s), and flagged data can be found in Supporting Table 1.

The time required to reach the ep is a function of both the time to draw the A_T_ down to zero at the anode plus the diffusion time required for the zone of neutralized A_T_ to reach the gate. The former is primarily controlled by the anode current, whereas the latter is influenced by both the anode current and anode–gate distance. The time required to reach the A_T_ titration ep decreased with increasing current as expected (Figure 3 and Table 1). For a single-constant anode current value, the ep decreases with A_T_ but even more so for the anode–gate distance where the three distinct groupings represent a change in the anode–gate distance. The slopes also show a trend in change where greater anode–gate distance and greater A_T_ show a greater change in ep versus current. Greater anode current corresponds to more proton evolution per unit time resulting in reaching the ep more rapidly at the anode. For greater the anode–gate distance, more time is required for the diffusion of the neutralized A_T_ zone, and there was less sensitivity to change in anode current as shown by the more similar slopes in Table 1 for the 150 and 200 μm configurations compared to 100 μm.

**Figure 3:**
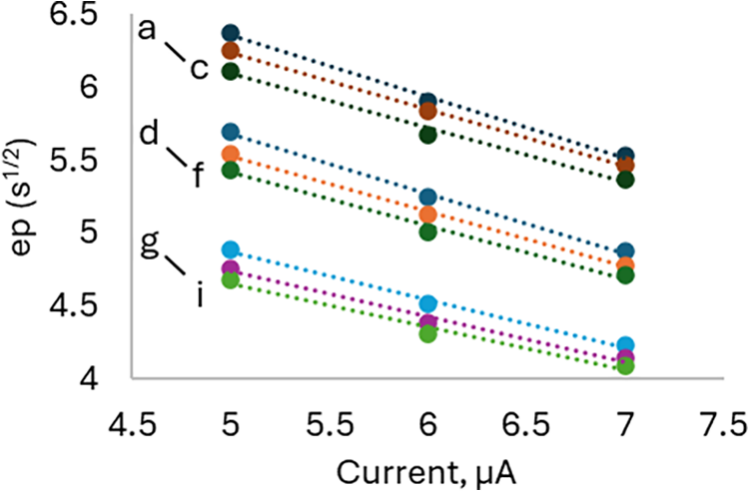
A_T_ ep derived by the CDT method versus the anode current for the 3 anode–gate distances and 3 A_T_ solutions. Starting from the top, (a–c) correspond to an anode–gate distance of 200 μm for A_T_ = 2264 (a), 2223 (b), and 2186 (c) μmol kg^–1^; (d–f) correspond to an anode–gate distance of 150 μm for A_T_ = 2264 (d), 2223 (e), and 2186 (f) μmol kg^–1^; and (g–i) correspond to an anode–gate distance of 100 μm for A_T_ = 2264 (g), 2223 (h), and 2186 (i) μmol kg^–1^. The slopes of the linear regressions are given in Table 1.

**Table 1:** Anode Current Sensitivity^a^

	2264 μmol/kg	2223 μmol/kg	2186 μmol/kg
200 μm	–0.418 (a)	–0.392 (b)	–0.372 (c)
150 μm	–0.409 (d)	–0.378 (e)	–0.362 (f)
100 μm	–0.328 (g)	–0.322 (h)	–0.294 (i)

a As determined by the slopes from Figure 3 (s^1/2^/μA).

Similar to Figure 3 and Table 1, Figure 4 and Table 2 present the relationship between ep and the anode–gate distance, where the sensitivity of ep determination is represented by the slope. Unlike the current, the anode–gate distance does not exhibit distinct groupings associated with A_T_. The slopes or change in ep versus anode–gate distance are nearly constant for a given anode current, with a small reduction at the lowest A_T_ value. Here, the smaller anode–gate distance corresponds to the shorter diffusion time required for the “reaction zone” to reach the gate, or the pH-sensing region, of the ISFET, resulting in reaching the ep more rapidly.

**Figure 4:**
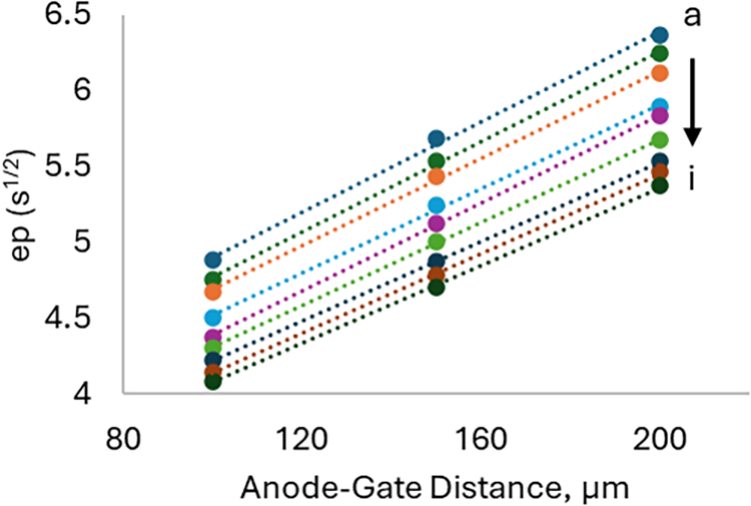
A_T_ ep derived by the CDT method versus the anode–gate distance for the 3 anode current levels and 3 A_T_ solutions. Starting from the top, (a–c) correspond to anode current of 5 μA for A_T_ = 2264 (a), 2223 (b), and 2186 (c) μmol kg^–1^; (d–f) correspond to anode current of 6 μA for A_T_ = 2264 (d), 2223 (e), and 2186 (f) μmol kg^–1^; and (g–i) correspond to anode current of 7 μA for A_T_ = 2264 (g), 2223 (h), and 2186 (i) μmol kg^–1^. The slopes of the linear regressions are listed in Table 2.

**Table 2:** Anode–Gate Distance Sensitivity^a^

	2264 μmol/kg	2223 μmol/kg	2186 μmol/kg
5 μA	0.0149 (a)	0.0149 (b)	0.0144 (c)
6 μA	0.0139 (d)	0.0145 (e)	0.0137 (f)
7 μA	0.0131 (g)	0.0132 (h)	0.0128 (i)

a As determined by the slopes from Figure 4 (s^1/2^/μm).

Figure 5 presents a combined view of all three experimental variables where linear regression between the ep and known solution A_T_ can be used to translate between ep (s^1/2^) and sensor A_T_. The slopes increased with increasing anode current and decreasing anode–gate distance as expected except in the 5 μA case where the slopes were very similar between 150 and 200 μm. *A low slope in* Figure 5 *corresponds to greater A_T_* *resolution*. Using 7 μA anode current resulted in a significant increase in slope or reduction in A_T_ resolution, and the optimal configuration was 5 μA, 150–200 μm in terms of A_T_ resolution. The linearity of the 6 μA, 200 μm configuration showed the lowest *R* ^2^ value, and this could be due to possible bubble disruption. There was greater standard deviation across the 15 measurements used in this linear regression compared to the other configurations.

**Figure 5:**
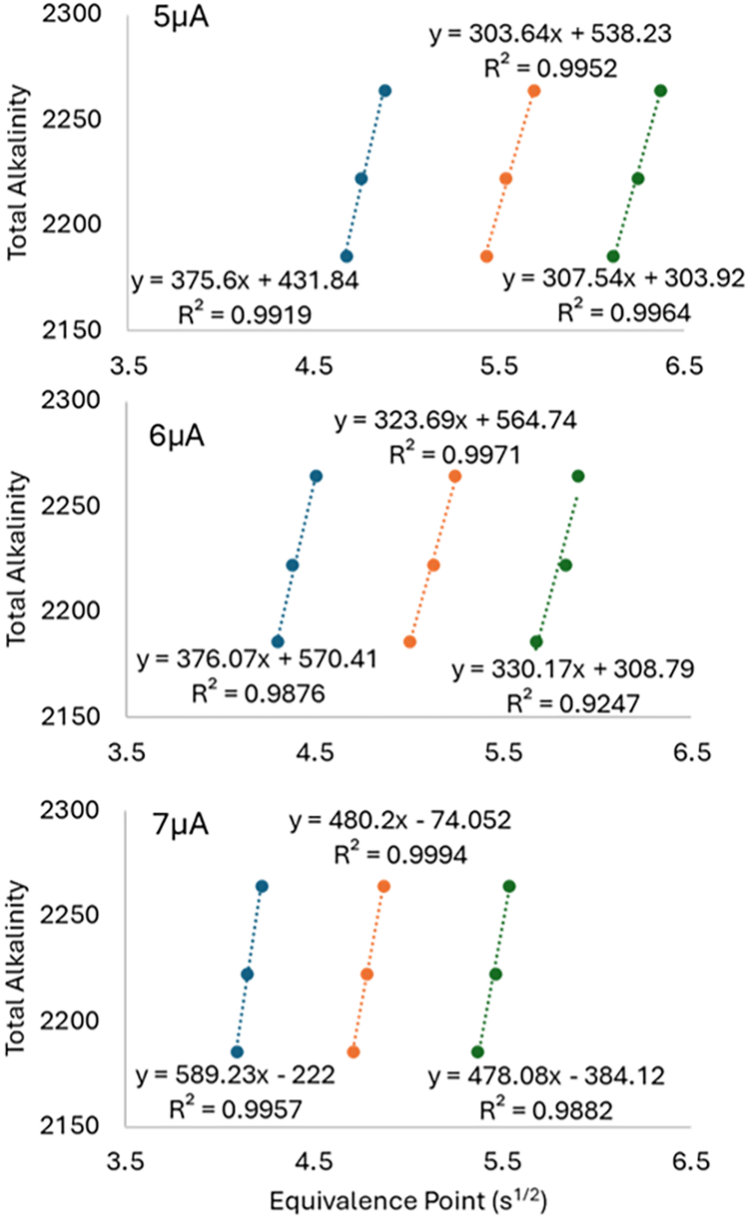
Dilution curves for each of the configurations of the anode–gate distance, anode current, and A_T_. The anode current was varied between 5 (top), 6 (middle), and 7 μA (bottom). The anode–gate distance was varied from 100 (leftmost, blue), 150 (middle, orange), and 200 μm (rightmost, green). The A_T_ was diluted from 2264 to 2223 to 2186 μmol kg^–1^ by adding DI water to the starting seawater solution by weight. The resulting linear regression (with *R* ^2^ shown) provides the conversion between CDT ep and A_T_.

The average root-mean-square error (RMSE) for the entire data series without averaging at each configuration was 9.90 μmol kg^–1^. When averaging the 5 data points (if not flagged as possible bubble disruption) collected at each permutation, the error was reduced to 2.85 μmol kg^–1^. The median error was 2.16 μmol kg^–1^ with a maximum and minimum of 12.1 and 0.46 μmol kg^–1^, respectively. See Supporting Table 1 for the full list of RMSE details.

## Discussion

Part of the necessity to explore new approaches different from the CDT method outlined in Briggs et al.^22^ (where the actuator electrode was deposited directly on the ISFET chip with a mechanical mask) was the difficulty in replication due to the occurrence of bubble production on the anode. In the initial work, a skilled machinist provided high-precision masks that were used in the electrode deposition process. When this process was repeated by others several years later, rough mask edges were introduced. This led to particulate sloughing from the mask during vacuum deposition, causing contamination on the exposed surface of the ISFET. Surface contaminants promote the formation of nodule islands during Pt deposition (Figure 6). The resulting electrode roughness decreases the energy required for bubble nucleation.^35,36^

**Figure 6:**
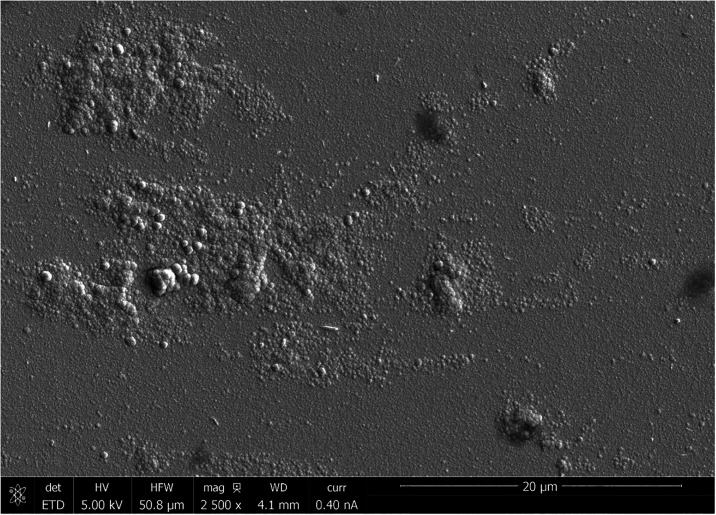
SEM image of the anode surface from the batch of modified ISFETs using defective masks showing nodule islands and increased surface roughness. Pt deposition by argon sputtering typically achieves an ultrasmooth surface. The previous batch of ISFETs modified with higher precision masks showed no surface features, nodules, or roughness.

When bubbles form on the electrode and stick to the ISFET (Figure 7), several new complications arise. First, the bubbles effectively reduce the total area of the electrode, which leads to an effective change in current density (increase) when a constant current is applied. Further bubble growth continues changing the current density until bubbles move or dislodge which causes microturbulences disrupting the diffusion-based CDT measurement. If a bubble crosses or sticks to the gate of the ISFET, then the Nernstian response is impacted. The other peculiarity with bubble production is that it is difficult to flush the bubbles off of the face of the ISFET. This results in low pH microenvironments in the presence of a gas bubble (presumably O_2_ or possibly Cl_2_ or even CO_2_ from acidified seawater), and over the duration of several hours, a precipitate film visually forms on the anode and spreads over the entire ISFET. The film changes the proton evolution efficiency and the Nernstian response of the ISFET. Elemental analysis of the film has been inconclusive as nothing was present in the analysis that was not already expected to be present within the ISFET material composition. The film can be removed with acetone if it has not been fully dried.

**Figure 7:**
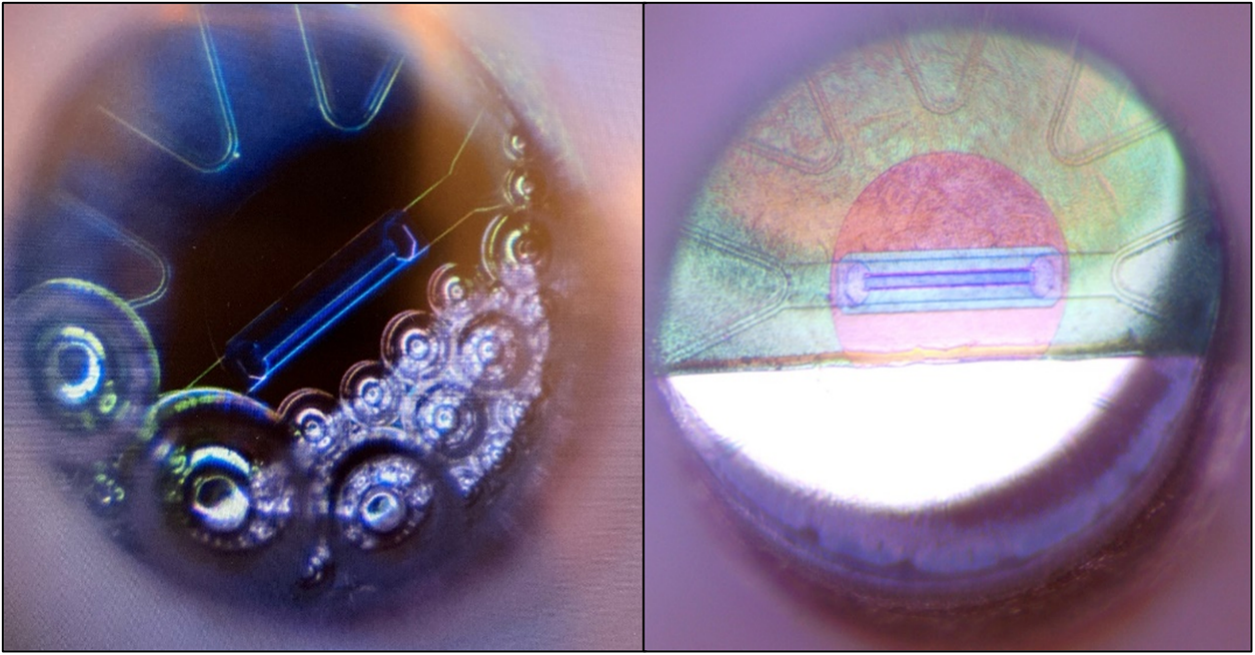
View of the region of the pH–A_T_ sensor exposed to solution with clear evidence of bubble generation on the anode (left) and discoloration due to film formation (right).

While other high-precision machining efforts could be explored to reduce the mask edge roughness, clearly, the back-end processing of a single die or small batch is labor-intensive and difficult to reproduce. Another key drawback is material “creep” under the masked zone during the anode deposition processes and potential interference with the pH functionality of the ISFET. With the approach presented here, all off-the-shelf supplies can be assembled to perform the same ISFET-based pH and A_T_ measurement based on the CDT method. Another key benefit is the ability to adjust the anode–gate distance “on the fly” with the same ISFET without making any other changes (i.e., swapping out sensors). Some bubble production was still evident in the early testing of the suspended anode assembly. A small scratch was identified on the initial anode that proved to be difficult to polish smooth. Replacing the electrode with a defect-free one helped in addition to increasing the flushing with more directed flow between titrations to facilitate the removal of a bubble if one formed so it would not linger and grow. Other electrode materials or methods for obtaining an ultrasmooth surface can be explored; however, the convenience of the readily available off-the-shelf electrode proved very effective after polishing, and we suspect bubbles to be less of an issue when operating the sensor in situ under even slightly elevated pressure.

An interesting aspect of the vertically suspended anode is that the diffusion zone is more unidirectional relative to the on-chip configuration (Figure 8). Within the small vertical gap immediately between the anode and the gate, diffusion can only propagate in the *z*-direction for a sufficiently large enough anode positioned close to the gate. In the on-chip anode case, diffusion is propagating in 3 dimensions away from the anode. With the suspended anode, we observed sharper inflections corresponding to the titrated seawater species when compared to similar measurements made with the on-chip anode likely due to differing diffusion dynamics (see Figure 9 compared to Figure 1). Because the gap between the anode and ISFET is small (100–200 μm), the pump ON-time had to be increased to ensure complete flushing. Integration of the anode into a microfluidic channel in the future will reduce the pump time to ∼10 s.

**Figure 8:**
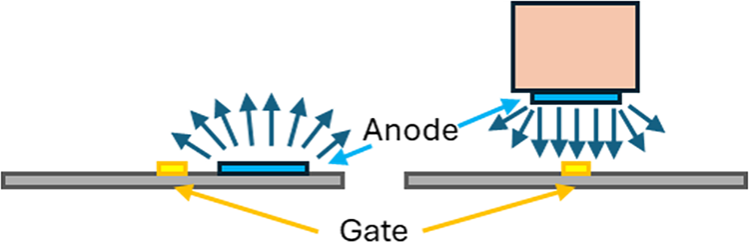
Depiction of the diffusion dynamics (dark blue arrows) for the on-chip (left) and suspended above-chip (right) anodes.

**Figure 9:**
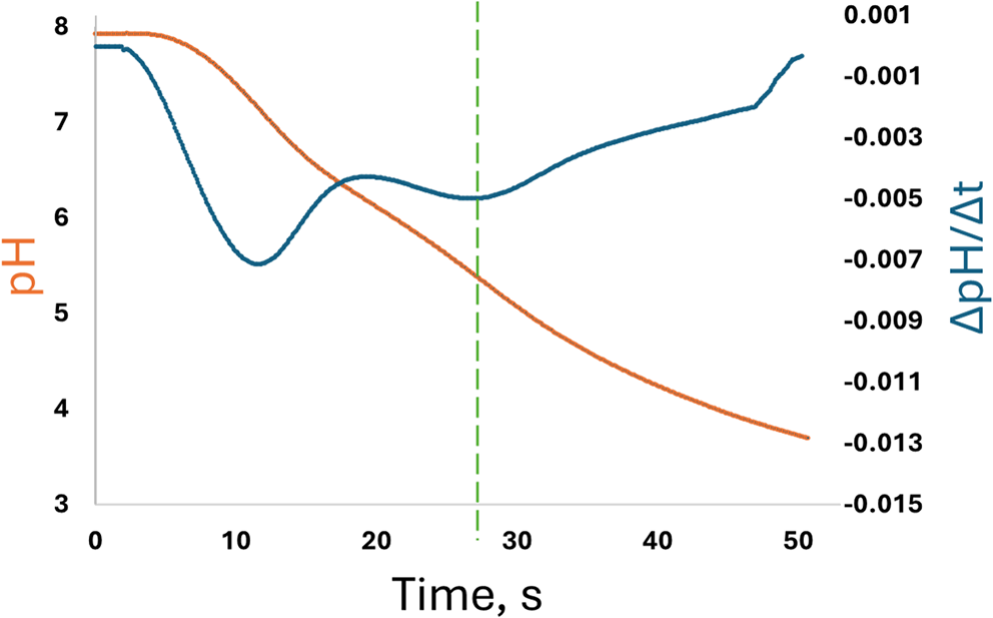
Example of a titration curve (orange) and first derivative (blue) collected by an ISFET with an on-chip anode under very similar operating conditions and set to the same scale as in Figure 1. The inflection corresponding to the ep (dashed green line) is much more diffused compared with the results with the new suspended anode approach.

With the demonstrated linearity of the CDT method over a large range of seawater A_T_ spanning <2100–2600 μmol kg^–1^ from the early work with the anode on the chip,^22^ we can extrapolate the required titration time for the configurations representing the upper and lower bounds (see Table 3).

**Table 3:** Extrapolated ep Time Requirements

	2600 μmol/kg	2000 μmol/kg
200 μm, 5 μA	56 s	30 s
100 μm, 7 μA	23 s	14 s

Based on the results presented here, the preferable configuration for maximizing A_T_ resolution proved to be a 5 μA anode current, with a 150–200 μm anode–gate distance. This configuration increases the duration of a single measurement by ∼10 s, which may be a consideration for applications where sensor response is of greater concern. With increased averaging, the precision improves but again at the expense of increased measurement time. For a smaller A_T_ range, it may be interesting to explore even larger anode–gate distance and/or lower anode current to test the limit of A_T_ resolution which will eventually diminish as the ep peak is smoothed out by diffusion.

The 135 measurements (27 configurations with 5 replicates each) used in this analysis required 6 h of continuous measurement to complete. This time frame makes replication on the same solution in a single experimental run challenging due to sample drift caused by evaporation. Future studies would benefit by completing multiple replications for a single configuration (to assess sensor stability over longer periods) and use of a microchannel to improve flushing.

Because the analytical signal (time) is a function of anode current density rather than current, consideration must be given to the sensor geometry. In this work, we used a circular 1 mm diameter Pt disc with an area of 0.785 mm^2^, so the current densities used in this experiment were 6.4, 7.6, 8.9 μA mm^–2^. An additional consideration is the proton evolution efficiency of the electrode material. Different manufacturers have different metal purity and surface roughness, which may translate to varying proton evolution efficiency even when operated at the same current density.

## Conclusions

Here, we demonstrated a reagentless, solid-state sensor using all off-the-shelf parts to measure pH and A_T_ of seawater. A micrometer-driven translational stage was used to position a titrant-generating electrode vertically above an ISFET to perform an acid–base titration. For a larger range of A_T_, a greater anode–gate distance may be desirable with a lower anode current to be able to resolve the ep over the full range. The greater the anode–gate distance and lower the anode current, the longer it takes to reach the ep for a given A_T_ and thus is more optimal for lower A_T_ determination. Conversely, the smaller the anode–gate distance and the greater the anode current, the shorter it takes to reach the ep for a given A_T_ and thus more optimal for higher A_T_ determination.

A logical direction for future development of the suspended anode approach is the incorporation of a microfluidic manifold with the anode precisely positioned at a static distance from the gate (no micrometer apparatus is required). Initial designs are underway and may offer the benefit of reducing sample volume, increasing flushing rate with directed flow through the anode–gate gap to facilitate bubble removal, and simplifying integration on in situ platforms. The smaller volume requirement of a microfluidic cell also opens opportunities for sampling small volume spaces in situ, such as porewaters. The influence of turbulent motion on the diffusion-based measurement may also be minimized in a microfluidic cell, although passive valves can easily be added to reduce externally induced motion in the cell. For environments with high organic or particulate load (e.g., coastal zones) the contribution of organic alkalinity can be a non-negligible component of the measured A_T_ and interference by particles will need to be addressed. It is feasible to filter intake water to reduce or eliminate the particles. Although beyond the scope of this work, we are developing an algorithm that may provide information on contributions of unknown proton acceptors associated with organic alkalinity, from the analysis of the entire titration curve including both inflection points.

Much characterization is still required prior to in situ deployment to understand the sensor response and to calibrate over the full range of T, S, and P in natural waters. While the sensitivity of the ISFET response to T, S, and P has been carefully characterized for in situ applications,^25^ in A_T_ mode, there is likely a nonzero effect on diffusion time and proton evolution efficiency that will require careful examination and calibration.

## Supplementary Material



## Notes

Supporting spreadsheet containing sensor configuration, titration data, calibration data, and RMSE details (CSV)

## Abbreviations

A_T_: total alkalinity
ISFET: ion-sensitive field-effect transistor
CDT: coulometric diffusion titration
ep: equivalence point

## References

[1] Gruber N., Gloor M., Mikaloff Fletcher S.
E., Doney S. C., Dutkiewicz S., Follows M. J., Gerber M., Jacobson A. R., Joos F., Lindsay K., Menemenlis D., Mouchet A., Müller S. A., Sarmiento J. L., Takahashi T. (2009). Oceanic Sources, Sinks, and Transport of Atmospheric
CO2. Global Biogeochem. Cycles.

[2] Gruber N., Bakker D. C. E., DeVries T., Gregor L., Hauck J., Landschützer P., McKinley G. A., Müller J. D. (2023). Trends
and Variability in the Ocean Carbon Sink. Nat.
Rev. Earth Environ..

[3] Wang Z. A., Moustahfid H., Mueller A. V., Michel A. P. M., Mowlem M., Glazer B. T., Mooney T. A., Michaels W., McQuillan J. S., Robidart J. C., Churchill J., Sourisseau M., Daniel A., Schaap A., Monk S., Friedman K., Brehmer P. (2019). Advancing Observation of Ocean Biogeochemistry,
Biology,
and Ecosystems With Cost-Effective in Situ Sensing Technologies. Front. Mar. Sci..

[4] Martz T. R., Daly K. L., Byrne R. H., Stillman J. H., Turk D. (2015). Technology
for ocean acidification research: Needs and availability. Oceanography.

[5] Bushinsky S. M., Takeshita Y., Williams N. L. (2019). Observing Changes in Ocean Carbonate
Chemistry: Our Autonomous Future. Curr. Clim.
Change Rep..

[6] Chai F., Johnson K. S., Claustre H., Xing X., Wang Y., Boss E., Riser S., Fennel K., Schofield O., Sutton A. (2020). Monitoring Ocean Biogeochemistry
with Autonomous Platforms. Nat. Rev. Earth Environ..

[7] Prien R. D. (2007). The Future
of Chemical in Situ Sensors. Mar. Chem..

[8] Millero F. J. (2007). The Marine
Inorganic Carbon Cycle. Chem. Rev..

[9] Dickson, A. G. ; Sabine, C. L. ; Christian, J. R. Guide to Best Practices for Ocean CO2 Measurements; North Pacific Marine Science Organization, 2007.

[10] Gray S. E. C., DeGrandpre M. D., Moore T. S., Martz T. R., Friederich G. E., Johnson K. S. (2011). Applications of in Situ PH Measurements
for Inorganic Carbon Calculations. Mar. Chem..

[11] Carter B. R., Sharp J. D., García-Ibáñez M. I., Woosley R. J., Fong M. B., Álvarez M., Barbero L., Clegg S. L., Easley R., Fassbender A. J., Li X., Schockman K. M., Wang Z. A. (2024). Random and Systematic Uncertainty
in Ship-Based Seawater Carbonate Chemistry Observations. Limnol. Oceanogr..

[12] Middelburg J. J., Soetaert K., Hagens M. (2020). Ocean Alkalinity,
Buffering and Biogeochemical
Processes. Rev. Geophys..

[13] Lee K., Tong L. T., Millero F. J., Sabine C. L., Dickson A. G., Goyet C., Park G.-H., Wanninkhof R., Feely R. A., Key R. M. (2006). Global Relationships
of Total Alkalinity
with Salinity and Temperature in Surface Waters of the World’s
Oceans. Geophys. Res. Lett..

[14] Millero F. J., Lee K., Roche M. (1998). Distribution
of Alkalinity in the Surface Waters of
the Major Oceans. Mar. Chem..

[15] Carter B. R., Bittig H. C., Fassbender A. J., Sharp J. D., Takeshita Y., Xu Y.-Y., Álvarez M., Wanninkhof R., Feely R. A., Barbero L. (2021). New and Updated Global
Empirical
Seawater Property Estimation Routines. Limnol.
Oceanogr.: Methods.

[16] Carter B. R., Williams N. L., Gray A. R., Feely R. A. (2016). Locally Interpolated
Alkalinity Regression for Global Alkalinity Estimation. Limnol. Oceanogr.: Methods.

[17] Jiang Z.-P., Tyrrell T., Hydes D. J., Dai M., Hartman S. E. (2014). Variability
of Alkalinity and the Alkalinity-Salinity Relationship in the Tropical
and Subtropical Surface Ocean. Global Biogeochem.
Cycles.

[18] Cross J. N., Mathis J. T., Bates N. R., Byrne R. H. (2013). Conservative and
Non-Conservative Variations of Total Alkalinity on the Southeastern
Bering Sea Shelf. Mar. Chem..

[19] Bates N. R., Michaels A. F., Knap A. H. (1996). Alkalinity
Changes in the Sargasso
Sea: Geochemical Evidence of Calcification?. Mar. Chem..

[20] National Academies of Sciences, Engineering, and Medicine . A Research Strategy for Ocean-Based Carbon Dioxide Removal and Sequestration; The National Academies Press: Washington, DC, 2022. 35533244

[21] Briggs E.
M., De Carlo E. H., Sabine C. L., Howins N. M., Martz T. R. (2020). Autonomous
Ion-Sensitive Field Effect Transistor-Based Total Alkalinity and PH
Measurements on a Barrier Reef of Ka̅ne’ohe Bay. ACS Earth Space Chem..

[22] Briggs E. M., Sandoval S., Erten A., Takeshita Y., Kummel A. C., Martz T. R. (2017). Solid State Sensor
for Simultaneous
Measurement of Total Alkalinity and PH of Seawater. ACS Sens..

[23] Bergveld P. (2003). Thirty Years
of ISFETOLOGY: What Happened in the Past 30 Years and What May Happen
in the next 30 Years. Sens. Actuators, B.

[24] McLaughlin K., Dickson A., Weisberg S. B., Coale K., Elrod V., Hunter C., Johnson K. S., Kram S., Kudela R., Martz T., Negrey K., Passow U., Shaughnessy F., Smith J. E., Tadesse D., Washburn L., Weis K. R. (2017). An Evaluation
of ISFET Sensors for Coastal PH Monitoring Applications. Reg. Stud. Mar. Sci..

[25] Johnson K. S., Jannasch H. W., Coletti L. J., Elrod V. A., Martz T. R., Takeshita Y., Carlson R. J., Connery J. G. (2016). Deep-Sea
DuraFET:
A Pressure Tolerant PH Sensor Designed for Global Sensor Networks. Anal. Chem..

[26] Saba G. K., Wright-Fairbanks E., Chen B., Cai W.-J., Barnard A. H., Jones C. P., Branham C. W., Wang K., Miles T. (2019). The Development
and Validation of a Profiling Glider Deep ISFET-Based PH Sensor for
High Resolution Observations of Coastal and Ocean Acidification. Front. Mar. Sci..

[27] Martz T. R., Connery J. G., Johnson K. S. (2010). Testing
the Honeywell Durafet for
Seawater PH Applications. Limnol. Oceanogr.:
Methods.

[28] Bresnahan P. J., Martz T. R., Takeshita Y., Johnson K. S., LaShomb M. (2014). Best Practices
for Autonomous Measurement of Seawater PH with the Honeywell Durafet. Methods Oceanogr..

[29] Olthuis W., Bergveld P. (1995). Integrated Coulometric Sensor-Actuator
Devices. Microchim. Acta.

[30] Olthuis W., Luo J., Van der
Schoot B. H., Bergveld P., Bos M., Van der
Linden W. E. (1990). Modelling of Non-Steady-State Concentration Profiles
at ISFET-Based Coulometric Sensor—Actuator Systems. Anal. Chim. Acta.

[31] Olthuis W., Luo J., van der Schoot B. H., Bomer J. G., Bergveld P. (1990). Dynamic Behaviour
of ISFET-Based Sensor-Actuator Systems. Sens.
Actuators, B.

[32] van
der Schoot B., Bergveld P. (1985). An ISFET-Based Microlitre Titrator:
Integration of a Chemical Sensor—Actuator System. Sens. Actuators.

[33] van
der Schoot B., van der Wal P., de Rooij N., West S. (2005). Titration-on-a-Chip,
Chemical Sensor–Actuator Systems from Idea to Commercial Product. Sens. Actuators, B.

[34] Wen X., Herdan J., West S., Kinkade D., Vilissova N., Anderson M. (2004). Application of Rapid,
Electrochemical Flash Titration
to Total Acidity and Alkalinity Determinations in Buffers, Foods,
and Beverages. J. AOAC Int..

[35] Angulo A., van der Linde P., Gardeniers H., Modestino M., Rivas D. F. (2020). Influence of Bubbles
on the Energy Conversion Efficiency
of Electrochemical Reactors. Joule.

[36] Kempler P. A., Coridan R. H., Luo L. (2024). Gas Evolution
in Water Electrolysis. Chem. Rev..

